# The effect of ketamine on cognition, anxiety, and social functioning in adults with psychiatric disorders: A systematic review and meta-analysis

**DOI:** 10.3389/fnins.2022.1011103

**Published:** 2022-11-24

**Authors:** Mattia Marchi, Federica Maria Magarini, Giacomo Galli, Federico Mordenti, Antonio Travascio, Daniele Uberti, Edoardo De Micheli, Luca Pingani, Silvia Ferrari, Gian Maria Galeazzi

**Affiliations:** ^1^Department of Biomedical, Metabolic and Neural Sciences, University of Modena and Reggio Emilia, Modena, Italy; ^2^Dipartimento ad Attività Integrata di Salute Mentale e Dipendenze Patologiche, Azienda USL-IRCCS di Reggio Emilia, Reggio Emilia, Italy; ^3^Department of Mental Health and Drug Abuse, Azienda Unità Sanitaria Locale (AUSL) Modena, Modena, Italy; ^4^Villa Igea Psychiatric Hospital, Modena, Italy

**Keywords:** ketamine, esketamine, cognition, anxiety, social functioning, depression

## Abstract

**Background:**

It has been shown that ketamine can improve suicidality and depression. Evidence for other dimensions of psychopathology is lacking. We undertook a systematic review to investigate the effect of ketamine on cognition, anxiety, quality of life, and social functioning in adults with psychiatric disorders.

**Methods:**

PubMed (Medline), Scopus, PsycINFO, and EMBASE were searched up to April 2022. Randomized controlled trials (RCTs) on ketamine [or its S (+) enantiomer] reporting data on cognition, anxiety, quality of life, social functioning in adults with psychiatric disorders were included. Standardized mean difference (SMD) was used for summarizing continuous outcomes.

**Results:**

Twenty-two reports were included in the final selection, of which 20, corresponding to 1,298 participants, were included in the quantitative synthesis. Affective disorders were the predominant diagnostic category. Median follow-up time was 21 days. The evidence was rated moderate to very low. In most trials, ketamine was administered intravenously or as adjuvant to electro-convulsant therapy (ECT). Only 2 trials of intranasal esketamine were identified. The effect of ketamine on depression was confirmed (SMD: −0.61 [95% CI: −1.06; −0.16]). Furthermore, by pooling results of 6 RCTs, ketamine may be effective in reducing anxiety symptoms (SMD: −0.42 [95% CI: −0.84; 0.003]), particularly when administered not within ECT (5 trials; SMD: −0.58 [95% CI: −1.07; −0.09]). However, there was moderate heterogeneity of results. Patients treated with ketamine also had an improvement in social functioning (SMD: −0.31 [95% CI: −0.52; −0.10]), although the estimate was based only on 2 studies. No difference to comparators was found with respect to cognition and quality of life.

**Conclusion:**

Alongside the antidepressant effect, ketamine may also improve anxiety and social functioning in adults with affective disorders.

## Introduction

Ketamine is a non-competitive N-Methyl-D-Aspartate Receptor (NMDAR) antagonist mainly used as a dissociative anesthetic agent (US Food Drug Administration, 1970). In the last few years, a growing body of evidence supported also its rapid antidepressant and anti-suicidal effect (Sanacora et al., 2017; Feder et al., 2020; Dean et al., 2021), leading in 2019 to the approval by the FDA of ketamine [in its enantiomeric S (+) form, esketamine] for the treatment of Treatment-Resistant Depression (TRD) in addition to an oral antidepressant (US Food Drug Administration, 2019). The rapidity of the antidepressant effect of sub-anesthetic ketamine is particularly important when compared to other treatments for depression, such as serotonin selective re-uptake inhibitors which are characterized by a latency to treatment response of several weeks (Gerhard et al., 2016). Instead, the antidepressant effects of ketamine typically become evident within a few hours or 1 day of a single infusion (Newport et al., 2015). After a single infusion of a sub-anesthetic dose of ketamine, the benefits generally disappear within 1 week, but repeated ketamine infusions have shown cumulative and sustained antidepressant effects, with reduction in depressive symptoms maintained through once-weekly infusions (Phillips et al., 2019).

Ketamine is also able to induce psychotic-like conditions (with hallucinations and delusions) and it is used in experimental models of psychosis (Beck et al., 2020; Marchi et al., 2021). The antidepressant and anti-suicidal effects of ketamine seem to be not exclusive to major depression disorder (MDD), rather they are played also across other affective disorders, including bipolar depression, anxiety disorders, and possibly also on depression with psychotic features, despite its relative contraindication in psychosis (Witt et al., 2020; Cavenaghi et al., 2021; Lima et al., 2022; Souza-Marques et al., 2022). Furthermore, there is increasing interest in the possible application of ketamine in other disorders, such as anxiety disorder, obsessive-compulsive disorder, and post-traumatic stress disorder (Whittaker et al., 2021; Jumaili et al., 2022).

The wide pharmacodynamic effects of ketamine is not limited to the NMDAR antagonism, but rather is exerted also through modulation of GABA, BDNF, opioids, and monoamine systems, and through its neuroactive metabolites which are still under investigation (Strzelecki et al., 2013; Zhou et al., 2014; Hess et al., 2022). This suggests on one hand to explore possible other therapeutic applications of the substance outside TRD, and on the other if there are other effects of ketamine that contribute to the improvement in TRD patients next to the antidepressant one.

NMDARs play an important role in neuro-cognition and neurotoxicity. Previous animal and human studies have suggested that, under certain conditions, ketamine is neurotoxic and that both short- and long-term use of ketamine may impair cognitive function, particularly in learning and memory tasks (Ding et al., 2016; Melo et al., 2016). The available literature suggests that short-term cognitive impairments follow a single sub-anesthetic dose of ketamine, while long-term impairments in the context of drug abuse are generally seen in individuals who utilize a much higher dose of ketamine than that commonly applied in clinical trials (Morgan et al., 2004, 2012). Furthermore, in a longitudinal study over 12 months, long-term memory impairments have been detected only in frequent high-dose ketamine users, suggesting that ketamine's negative cognitive effects may be reversible at lower doses and less frequent administrations (Morgan et al., 2010). Addressing ketamine's cognitive effects is important as cognitive dysfunction is also recognized as one of the symptoms of major depressive disorder and TRD (Bortolato et al., 2014; Knight and Baune, 2018). From a biochemical perspective, ketamine's antidepressant effects are more commonly viewed within a “cascade” framework of intracellular events that move far beyond NMDAR blockade of GABAergic interneurons and are thought to rapidly promote neuroplasticity. Evidence from both animal and human studies, support the importance of this “cascade” for sub-anesthetic doses of ketamine which can promote synaptogenesis and neuroplasticity in several brain areas, such as the medial prefrontal cortex (mPFC) and some limbic regions (Deyama and Duman, 2020). These areas are associated with specific cognitive functions such as verbal fluency, strategic planning and organization, as well as attention and concentration, and can be altered in depressed patients (Price and Duman, 2020). By inhibiting GABAergic interneurons, ketamine generates both a rapid burst of glutamate and AMPA receptor activation with immediate release of BDNF and VEGF (Deyama and Duman, 2020). The binding of BDFN and VEGF to their respective targets (i.e., the TrkB and Flk-1 receptors) activates the mammalian target of rapamycin (mTOR) intracellular pathway mTORC1 which fosters the expression of presynaptic and postsynaptic proteins—-such as Synapsin1, PSD95, and GluR1 - important for neural spine maturation and synaptic strengthening (Li et al., 2010). Eventually, this translates into a better cortico-limbic connection, potentially improving overall cognitive functioning, especially in people with depression.

Anxiety is often comorbid with depressive disorders, with estimates of the overlap ranging from 45 to 67% (Fava et al., 2008; Lamers et al., 2011). Low-dose ketamine has been mainly studied in treatment-resistant depression, with fewer reports on anxiety disorders. However, it has been demonstrated that glutamate and NMDARs are involved in the stress response and fear extinction (Davis and Myers, 2002) and changes in the glutamate pathway have been linked to the development of anxiety disorders (Freitas-Ferrari et al., 2010; Kormos and Gaszner, 2013). A recent meta-analysis suggests that ketamine may also have significant anxiolytic effects on patients with anxiety spectrum disorders, including treatment-refractory cases (Whittaker et al., 2021).

Finally, quality of life and social functioning may be relevant outcomes in depression and other psychiatric disorders since these usually come with a high personal and social burden. For example, depression is listed among the leading causes of disability worldwide, as measured by both Years Lived with Disability (YLDs) and disability-adjusted life-year (DALY) metrics (GBD 2019 Mental Disorders Collaborators, 2022). People suffering from mental health problems experience high levels of social impairment and lower quality of life, which improvement is one of the main challenges during the treatment.

The aim of this systematic review was to address the antidepressant effect of ketamine across different psychiatric disorders and to address the effect of ketamine on specific trans-diagnostic domains of psychopathology. Given the background highlighted above, we choose a priori to focus on cognition, anxiety, and indicators of disability (i.e., quality of life and social functioning).

## Methods

The protocol of this systematic review was registered with PROSPERO (CRD42022325534).

### Search strategy and inclusion/exclusion criteria

We searched the PubMed (Medline), Scopus, PsycINFO, and EMBASE databases until April 30, 2022, using the strategy outlined in the Supplementary Table 1 of the Appendix. No restrictions regarding language of publication or publication date were set. All RCTs comparing ketamine or esketamine used as monotherapy or as add-on treatment to placebo or other active comparators in adults (aged 18 years or above) with any psychiatric disorders were eligible for the review. Diagnosis was defined according to standard operational diagnostic criteria (according to the *Diagnostic and Statistical Manual of Mental Disorders* [DSM] or the *International Classification of Diseases [ICD]*). Studies were excluded if the PICOS did not fit with that defined in the review protocol in PROSPERO (CRD42022325534). Specifically, studies that considered a sample of healthy volunteers or adults diagnosed only with substance use disorder were excluded, although substance use disorder was allowed as a comorbidity to another psychiatric disorder; studies where all participants received at least one dose of ketamine, or that did not provide post-treatment data on the outcomes considered for this review were also excluded. No other limits on participants' characteristics, concurrent treatment, or comorbidity were set. If data from the same trial were published in multiple papers, we considered only the publication reporting more complete information or, in case of parity in this criterion, the largest sample size, to maximize the power of the analyses. Sample overlap was ruled out through a careful check of the trial registration codes as well as the place and year(s) of sampling.

### Data collection and extraction

All retrieved articles in the original search were screened independently by three review authors (M.M., G.G., and F.M.M.) for inclusion, first on the title, followed by the abstract. This initial screening was followed by the analysis of full texts to check compliance with inclusion/exclusion criteria: the review authors were grouped into two groups, and each group independently screened full texts identifying studies for inclusion and recorded reasons for exclusion. All disagreements were explored until consensus was reached, and if consensus was not possible, another member of the team was consulted (G.M.G.).

For each eligible trial, the two groups of review authors independently extracted the following information: (1) Study characteristics (first author last name, year of publication, country, study setting, eligibility criteria, number of participants randomized in each arm, number of participants with outcome assessment); (2) Participant characteristics (age, sex, psychiatric diagnoses and stage of illness, symptoms severity at baseline, on-going psychiatric treatment); (3) Intervention details (comparator used, prescribed dosage and range, frequency of administration, route of administration, co-interventions); (4) Outcome measures of interest and time of data collection. Extraction sheets for each study were cross-checked for consistency and any disagreement was resolved by discussion within the research group.

### Outcome measures

The primary outcome was mean change in cognition, assessed through validated psychometric tools. Where reported, we also extracted data on the following secondary outcomes, all measured using validated scales: anxiety, social functioning, quality of life, depression, safety and tolerability (i.e., drop out due to any cause, drop out due to severe adverse effects, overall adverse effects, and death).

### Statistical analyses

Where possible, we summarized quantitative data among studies using meta-analyses. We used inverse-variance models with random effects to summarize both continuous and dichotomous outcome data (DerSimonian and Laird, 1986). For continuous outcome data, we calculated the Hedges' g standardized mean differences (SMDs) and the corresponding 95% confidence intervals (CIs); for dichotomous outcome data, we calculated the pooled odds ratios (ORs) and the corresponding 95% CIs (Cohen, 1988; Higgins et al., 2020). We used data from the intention-to-treat analyses for both continuous and dichotomous outcomes. The results were summarized using forest plots. Standard *Q* tests and the *I*^2^ statistic (i.e., the percentage of variability in prevalence estimates attributable to heterogeneity rather than sampling error or chance, with values of *I*^2^ ≥ 75% indicating high heterogeneity) were used to assess between-study heterogeneity (Higgins and Thompson, 2002; Higgins et al., 2021).

When the meta-analysis included at least 10 studies (Sterne et al., 2011), we performed funnel plot analysis and the Egger test to test for publication bias. If analyses showed a significant risk of publication bias, we would use the trim and fill method to estimate the number of missing studies and the adjusted effect size (Duval and Tweedie, 2000; Sutton et al., 2000; Terrin et al., 2003; Sterne et al., 2008). Meta-regression analysis was performed to examine sources of between-study heterogeneity on a range of study prespecified characteristics (i.e., depression effect size, length of follow-up, use as add-on or monotherapy, sex, age, and treatment resistance). The analyses were performed using *meta* and *metafor* packages in R (Balduzzi et al., 2019; RStudio Team, 2021; Schwarzer, 2021). Statistical tests were 2-sided and used a significance threshold of *p* < 0.05.

### Risk of bias assessment and the GRADE

Bias risk in the included studies was independently assessed by three reviewers (A.T., E.D.M., and D.U.), using the Cochrane risk of bias tool (Higgins et al., 2011). All disagreements were discussed until consensus, and if necessary, another member of the team was consulted (G.M.G.). Each item on the risk of bias assessment was scored as high, low, or unclear, and the GRADE tool was used to assess the overall certainty of evidence (Schünemann et al., 2013). Further information is available in the Supplementary Appendix.

## Results

### Study characteristics

**Figure 1:**
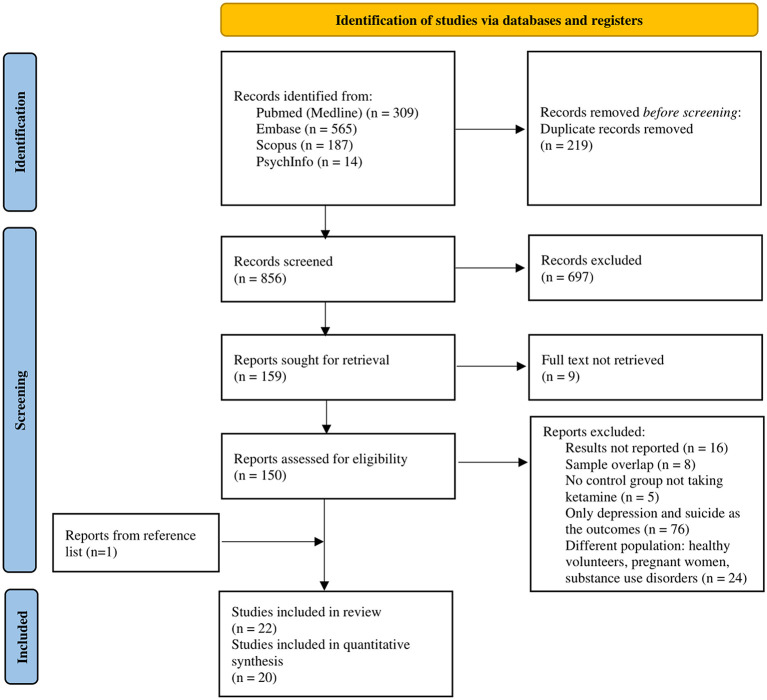
Preferred Reporting Items for Systematic Reviews and Meta-analyses (PRISMA) flow diagram.

As shown in Figure 1, from 856 records screened on title and abstract, 150 full texts were analyzed. The review process led to the selection of 22 studies (references reported in Table 1) referring to 22 independent RCTs (Loo et al., 2012; Zarate et al., 2012; Price et al., 2014; Rasmussen et al., 2014; Yoosefi et al., 2014; Alizadeh et al., 2015; Murrough et al., 2015; Singh et al., 2016; Zhong et al., 2016; Anderson et al., 2017; Fernie et al., 2017; Ray-Griffith et al., 2017; Chen et al., 2018; Taylor et al., 2018; Zhang et al., 2018; Dong et al., 2019; Fedgchin et al., 2019; Kheirabadi et al., 2019; Domany et al., 2020; Ochs-Ross et al., 2020; Keilp et al., 2021; Zou et al., 2021), 20 of these trials provided quantitative outcome data, therefore were included in the quantitative synthesis.

**Table 1:** Characteristics of the included studies.

**Author, year (Trial N)**	**Country**	**Diagnosis**	**Study design**	**Use**	**Days follow-up**	**N treatment (% females)**	**N control (% females)**	**Mean age (SD) treatment**	**Mean age (SD) control**	**Treatment**	**Control**
Alizadeh et al. (2015) (IRCT1388110 22935N2)	Iran	MDD	RCT	Anesthesia for ECT	6 ECT sessions	22 (72.7%)	20 (65%)	34.3 (10.7)	35.1 (12.4)	Ketamine (0.3 mg/kg) + Propofol + ECT	PBO + Propofol + ECT
Anderson et al. (2017) (ISRCTN14689382/ EudraCT 2011-005476-41)	UK	Unipolar and Bipolar Depression	RCT	Anesthesia for ECT	28	33 (66.7%)	37 (59.5%)	52.2 (11.9)	56.4 (12.4)	Ketamine (0.5 mg/kg) + ECT	PBO + ECT
Chen et al. (2018) (NR)	Taiwan	TRD	RCT	IV therapy	14	24 (87.5%)	24 (62.5%)	48.5 (11.0)	48.6 (8.1)	Ketamine 0.5 mg/kg	PBO
Domany et al. (2020) (NCT01887990)	US	Depression (any type)	RCT	IV therapy	3	9 (65.6%)	9 (65.6%)	35.1 (8.7)	35.8 (9.9)	Ketamine 0.2 mg/kg	PBO
Dong et al. (2019) (NCT02305394)	China	MDD	RCT	Anesthesia for ECT	28	43 (58.1%)	45 (51.1%)	36.8 (15.1)	35.7 (12.8)	Ketamine (0.3 mg/kg) + ECT	PBO + ECT
Fedgchin et al. (2019) (NCT02417064)	US	MDD	RCT	Intranasal spray therapy	28	115 (70.4%)	113 (71.7%)	46.4 (11.2)	46.8 (11.4)	Esketamine (56 mg) + AD	PBO + AD
Fernie et al. (2017) (NCT01306760)	UK	MDD	RCT	Anesthesia for ECT	28	20 (55%)	20 (55%)	51.8 (10.0)	49.9 (12.5)	Ketamine (0.5–1 mg/kg) + ECT	Propofol + ECT
Keilp et al. (2021) (NCT01700829)	US	MDD	RCT	IV therapy	1	39 (56.4%)	39 (64.1%)	37.2 (12.9)	39.6 (13)	Ketamine 0.5 mg/kg	Midazolam 0.02 mg/kg
Kheirabadi et al. (2019) (IRCT201104092266N2)	Iran	MDD	RCT	IV therapy	28	16 (25%)	15 (33.3%)	41.7 (12.9)	36.4 (14.1)	Ketamine 0.5 mg/kg	Thiopental (3 mg/kg) + ECT
Loo et al. (2012) (NCT00680433)	Australia	MDD	RCT	Anesthesia for ECT	28	22 (50%)	24 (70.8%)	45.2(15.6)	41.4(12.0)	Ketamine (0.5 mg/kg) + ECT	PBO + ECT
Murrough et al. (2015) (NCT01507181)	US	Transdiagnostic Suicidality	RCT	IV therapy	7	12 (66.7%)	12 (66.7%)	45.8 (15.2)	39.1 (10.6)	Ketamine 0.5 mg/kg	Midazolam
Ochs-Ross et al. (2020) (NCT02422186)	US	TRD	RCT	Intranasal spray therapy	28	72 (62.5%)	65 (61.5%)	70.6 (4.8)	69.4 (4.2)	Esketamine 28–84 mg + AD	PBO + AD
Price et al. (2014) (NCT00768430)	US	TRD	RCT	IV therapy	1	36 (56%)	21 (48%)	48.6 (11.4)	43.8 (10.9)	Ketamine 0.5 mg/kg	Midazolam 0.045 mg/kg
Rasmussen et al. (2014) (NR)^a^	US	Depression (any type)	RCT	Anesthesia for ECT	6 ECT sessions	21 (71.2%)	17 (47.1%)	47.0 (13.2)	48.6 (7.2)	Ketamine (1.05 mg/kg mean) + ECT	Methohexital (1.04 mg/kg mean) + ECT
Ray-Griffith et al. (2017) (NR)	US	Unipolar and Bipolar Depression	RCT	Anesthesia for ECT	21	8 (75%)	8 (87.5%)	43.6 (14.6)	38.1 (13.9)	Ketamine (1 mg/kg mean) + ECT	Methohexital (1 mg/kg) + ECT
Singh et al. (2016) (NCT01640080)	US	TRD	RCT	IV therapy	3	11 (64%)	10 (60%)	41.8 (11.6)	42.7 (10.9)	Ketamine 0.4 mg/kg	PBO
Taylor et al. (2018) (NCT02083926)	US	SAD	RCT crossover	IV therapy	14	9 (22.2%)	9 (55.6%)	30.78 (13.5)	28.67 (8.7)	Ketamine 0.5 mg/kg	PBO
Yoosefi et al. (2014) (IRCT201201247202N3)^a^	Iran	MDD	RCT	Anesthesia for ECT	28	17 (41.2%)	14 (46.7%)	40.9 (NR)	47 (NR)	Ketamine (1–2 mg/kg) + ECT	Thiopental (2–3 mg/kg) + ECT
Zarate et al. (2012) (NCT00088699)	US	Bipolar Depression	RCT crossover	IV therapy	1	40 (NR)	38 (NR)	46.7 (10.4)	46.7 (10.4)	Ketamine 0.5 mg/kg + Lithium or Valproate	PBO + Lithium or Valproate
Zhang et al. (2018) (NR)	China	Unipolar and Bipolar Depression	RCT	Anesthesia for ECT	28	43 (55.8%)	34 (50%)	31.47 (11.5)	28.6 (8.1)	Ketamine (0.5 mg/kg) + Propofol + ECT	Propofol + ECT
Zhong et al. (2016) (NR)	China	TRD	RCT	Anesthesia for ECT	21	30 (53.3%)	30 (66.7%)	32.1 (9.9)	29.2 (8.0)	Ketamine (0.8 mg/kg) + ECT	Propofol + ECT
Zou et al. (2021) (ChiCTR1800015082)	China	Depression (any type)	RCT	Anesthesia for ECT	28	76 (56.5%)	81 (58%)	65.76 (4.0)	65.6 (3.9)	Ketamine (0.3 mg/kg) + Propofol + ECT	PBO + Propofol + ECT

NR, not reported; UK, United Kingdom; US, United States of America; MDD, major depressive disorder; TRD, treatment-resistant depression; PTSD, post-traumatic stress disorder; SAD, social anxiety disorder; RCT, randomized controlled trial; ECT, electroconvulsive therapy; IV, intravenous; AD, antidepressant; TIMBER, trauma interventions using mindfulness-based extinction and reconsolidation; PBO, placebo.

a Not included in the quantitative synthesis.

The trials were all published in the last 10 years and were conducted in 6 countries: US (*n* = 11; 50.0%), China (*n* = 4; 18.2%), UK and Iran (each *n* = 2; 9.1%%), Australia and Taiwan (each *n* = 1; 4.5%). A total of 1,367 participants (718 treated with ketamine and 685 controls) were included in the review and 1,298 (680 ketamine and 654 controls) in the quantitative synthesis. The overall percentage of females across the studies was 56% (ketamine 55.8%; control 53.3%), mean age was 43.5 (SD = 9.7) years (ketamine 44.0 [SD = 9.8]; control 43.5 [SD = 10.2]). Almost all the studies (*n* = 20; 90.9%) involved patients with depression or suicidal ideation, and the most common psychiatric diagnosis was MDD (*n* = 8; 36.3%), followed by TRD (*n* = 5; 22.7%). One trial involved participants with social anxiety disorder (SAD, *n* = 1; 4.5%). In most of the studies ketamine was administered intravenously (IV), as adjuvant to ECT (*n* = 11; 50.0%) or as the single therapeutic agent (*n* = 9; 40.9%); dosing ranged from 0.2 to 2 mg/kg (median 0.5 mg/kg). Only 2 studies (9.1%) investigated the effect of intranasal esketamine (at a flexible dose ranging from 28 to 84 mg) as add-on to the current antidepressant treatment. The most common comparator was placebo (*n* = 12; 54.5%), followed by midazolam (*n* = 3; 13.6%). The main characteristics of the studies included in the review are summarized in Table 1.

Of the included studies, 17 trials, involving 1,209 participants (611 ketamine and 598 controls) provided quantitative outcome data on depression. As can be seen in Supplementary Figure 1, the meta-analysis yielded significant results supporting the efficacy of ketamine in improving depression (SMD: −0.61 [95% CI: −1.06; −0.16]; *p* = 0.008), although the estimate was affected by marked heterogeneity (*I*^2^ = 92%; *p* < 0.001) and important outlier effect played by the study from Zhong et al. (2016).

### Effect of ketamine on cognition

Sixteen RCTs investigated the effect of ketamine on cognition among people with depression.

**Figure 2:**
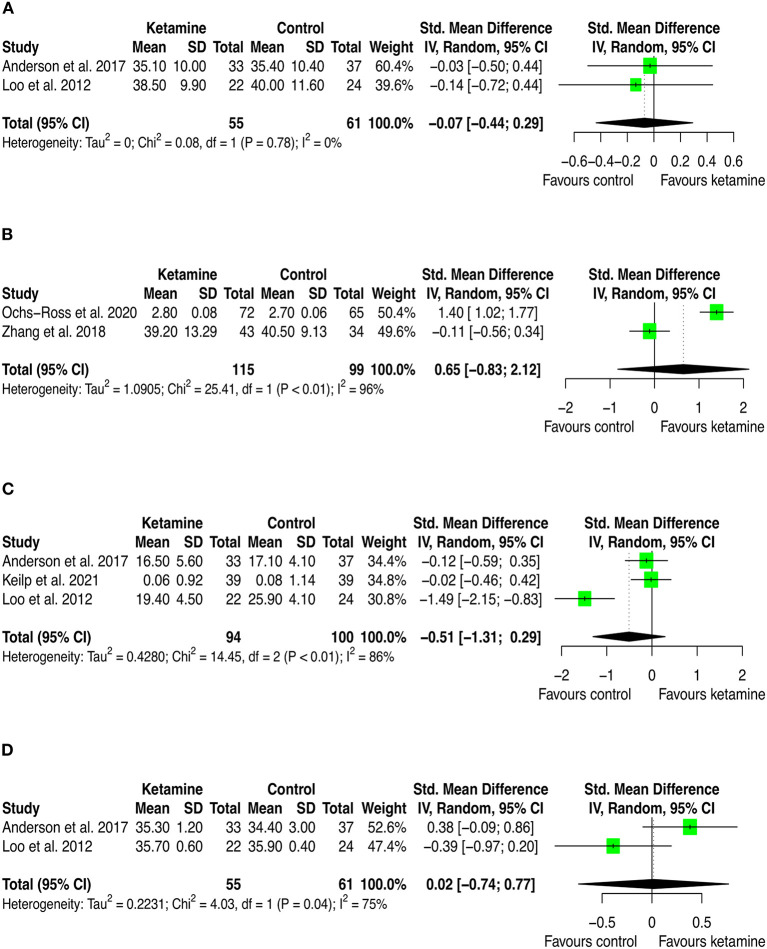
Forest plots of performance comparisons among experimental and control groups on autobiographical memory interview short form (AMI-SF) **(A)**, Attention **(B)**, Category fluency **(C)**, Copy **(D)**. SD, standard deviation; IV, inverse variance; 95% CI, 95% confidence interval. x axis labels have been edited according to the characteristics of the outcome: since higher scores indicate better performance the label “Favors ketamine” is on the right-hand side.

**Figure 3:**
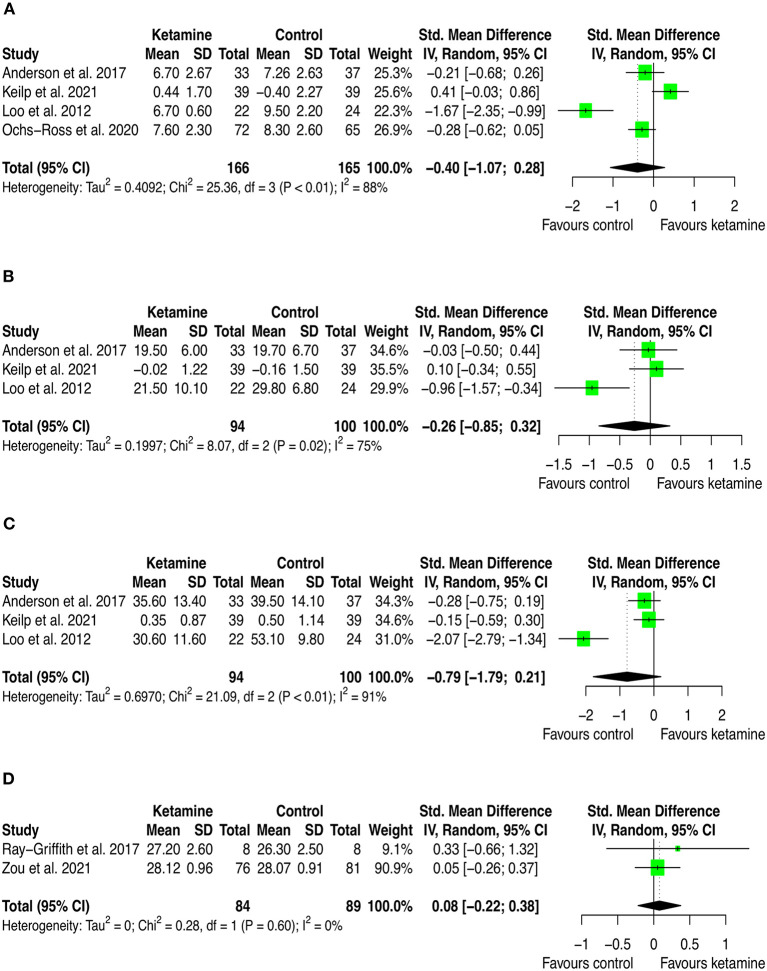
Forest plots of performance comparisons among experimental and control groups on Delayed recall **(A)**, Immediate recall **(B)**, Letter fluency **(C)**, Mini mental state examination (MMSE) **(D)**. SD, standard deviation; IV, inverse variance; 95% CI, 95% confidence interval. x axis labels have been edited according to the characteristics of the outcome: since higher scores indicate better performance the label “Favors ketamine” is on the right-hand side.

**Figure 4:**
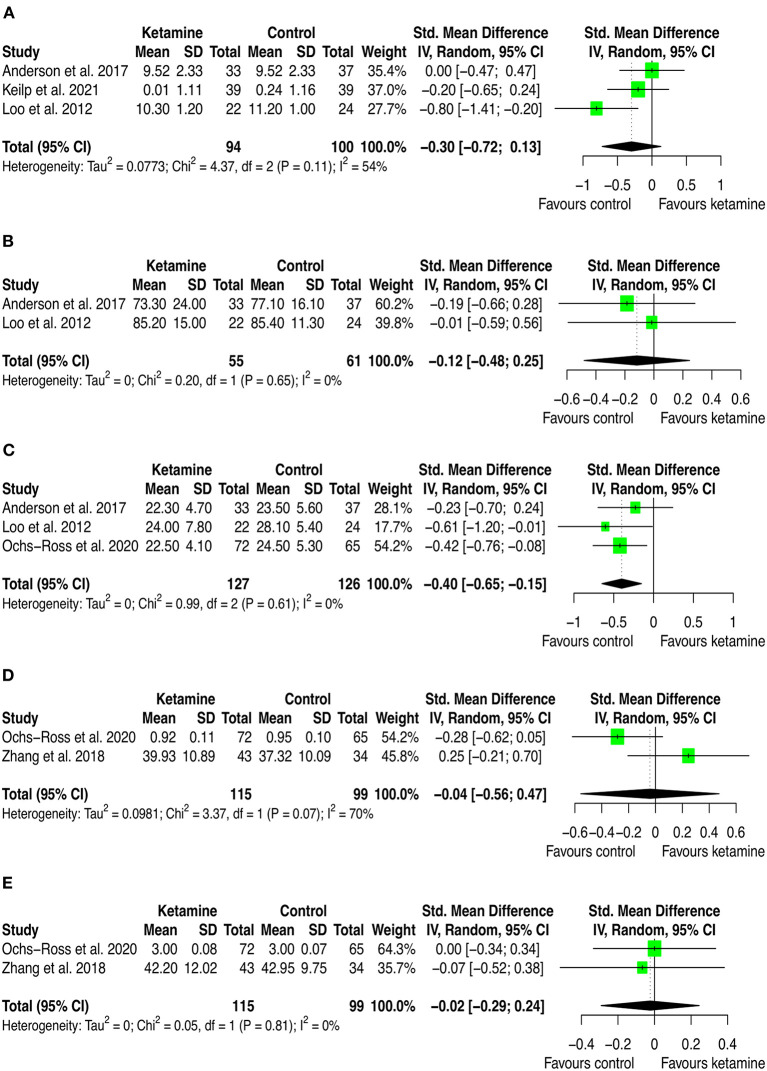
Forest plots of performance comparisons among experimental and control groups on Recognition discrimination **(A)**, Retention **(B)**, Total learning **(C)**, Visual learning **(D)**, Working memory **(E)**. SD, standard deviation; IV, inverse variance; 95% CI, 95% confidence interval. x axis labels have been edited according to the characteristics of the outcome: since higher scores indicate better performance the label “Favors ketamine” is on the right-hand side.

Twelve studies provided quantitative data that have been included in the meta-analysis. Of these, 9 (75.0%) trials used iv ketamine as adjuvant to ECT, 2 (16.6%) used IV ketamine as monotherapy, and one (8.3%) used intranasal esketamine added-on the current antidepressant medication. The comparator was either inactive (i.e., placebo [*n* = 6; 50.0%], nothing [*n* = 1; 8.3%]) or active agents (i.e., propofol [*n* = 2; 16.6], midazolam [*n* = 1; 8.3%], methohexital [*n* = 1; 8.3%], or ECT [*n* = 1; 8.3%]). Cognition was measured using validated cognitive battery of tests, and results were presented as the mean score on each cognitive dimension across the treatment and control arms. Therefore, we extracted outcome data for all the cognitive dimensions investigated and conducted one meta-analysis for each dimension which has been investigated in at least two studies. This led to 20 pairwise comparisons (presented Supplementary Table 2) and 13 distinct meta-analyses (see Figures 2–4). Among these, there was no significant difference between groups with isolated significant disadvantage for the ketamine-treated group in the total learning performance measured by the Hopkins Verbal Learning Test-Revised (HVLT-R-DR) (SMD: −0.40 [95% CI: −0.65; −0.15]; *p* = 0.002), although this estimate is based on only three studies.

By looking at the contribution of each study in these analyses, we can anyway detect interesting patterns. First, from a frequentist perspective all but two studies have consistently SMD estimates crossing zero. The two studies that provided significant individual estimates were Loo et al. (2012) and Ochs-Ross et al. (2020). The former included participants with depression and used ketamine in combination with ECT, the latter used esketamine as augmentation to oral antidepressant treatment for people with TRD. As stated in the introduction, ketamine's negative cognitive effects are thought to be dose-dependent and mainly impacting on memory and learning domains, with virtually no effects on attention (Morgan et al., 2004). This has been replicated in our analyses, where is possible to see that the effect on attention found by Ochs-Ross et al. (2020) favors esketamine, whereas the effect on total learning favors placebo. Also, Loo et al. (2012) which used ketamine as anesthetic within ECT session, detected consistently worse cognitive performance in the treatment group than in controls. That may be due both to the higher doses of ketamine implemented for anesthesia (the authors reported to have used 0.5 mg/kg IV ketamine) and to ECT which is known to negatively impact cognitive function. Given that all included studies that used ketamine in combination with ECT have administered ECT also to controls, it could be quite safe to attribute the negative effects on cognition to ketamine. Yet, one could also speculate that within a ECT session, ketamine and ECT may have a negative synergic effect on cognition. Further studies are needed to settle this argument.

Furthermore, there were 4 trials that assessed cognition in ketamine and control groups without providing quantitative data.

Chen et al. (2018) evaluated the effect of IV ketamine treatment on depression and cognition by enrolling and randomizing 71 TRD patients to 0.5 mg/kg ketamine, 0.2 mg/kg ketamine, or normal saline infusion groups. Cognition was measured through working memory task and a go/no go task at baseline, at day 3, and day 14 post-treatment administration. The authors concluded that a low dose of ketamine infusion did not impair cognitive function, but specific cognitive improvement in the sustained attention and response control (i.e., the go/no-go task) was observed only among the responders from the 0.5 mg/kg ketamine infusion group. In addition, the improvement was inversely proportional to depressive symptoms in the 0.5 mg/kg ketamine infusion group, suggesting that the antidepressant effect of ketamine infusion improves cognitive function.

Rasmussen et al. (2014) and Yoosefi et al. (2014) randomly assigned patients with depression candidate to ECT to receive anesthesia with either ketamine (21 and 15 patients, respectively), or active comparators (i.e., methohexital [17 patients], thiopental [14 patients], respectively). In both trials cognition was assessed with MMSE at baseline and after 6 ECT sessions. Rasmussen et al. did not find significant difference in the scores across the two groups after the treatment, whereas Yoosefi et al. reported significant difference favoring ketamine.

Singh et al. (2016) conducted a multicenter, randomized, placebo-controlled trial on 30 patients with TRD. Participants were randomly assigned to receive an IV infusion of 0.20 mg/kg (*n* = 9), 0.40 mg/kg esketamine (*n* = 11), or placebo (*n* = 10). The authors used the Massachusetts General Hospital-Cognitive and Physical Functioning Questionnaire (MGHCPFQ) to measure cognition and found that esketamine treatment (at any of the two tested doses) was associated with improvement of cognitive and physical functioning.

### Effect of ketamine on anxiety

The meta-analysis of the effect of ketamine on anxiety included 6 studies, involving 431 participants (223 ketamine and 209 controls). Participants were diagnosed with depression in 4 studies (66.7%), social anxiety disorder in 1 study (16.7%), and selected based on suicidal intent (regardless of the diagnosis) in 1 study (16.7%). In 4 studies (66.7%) the intervention was ketamine IV as monotherapy, 1 study (16.7%) used intranasal esketamine as add-on to the current antidepressant treatment, and 1 study (16.7%) used IV ketamine as adjuvant to ECT. The comparator was placebo for 4 studies (66.7%) and midazolam for 2 studies (33.3%). Anxiety was measured using the following validate instruments: Clinical Anxiety Scale (CAS), Beck Anxiety Index (BAI), Generalized Anxiety Disorder 7-items version (GAD-7), Concise Associated Symptoms Tracking (CAST) anxiety score, State-Trait Anxiety Inventory (STAI-S), Liebowitz Social Anxiety Score (LSAS).

**Figure 5:**
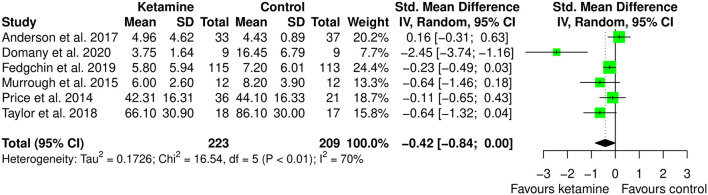
Forest plots of anxiety among ketamine/esketamine and control groups. SD, standard deviation; IV, inverse variance; 95% CI, 95% confidence interval. x axis labels have been edited according to the characteristics of the outcome: since lower scores indicate less anxiety the label “Favors ketamine” is on the left-hand side.

As can be seen in Figure 5 and Supplementary Table 3, ketamine can improve anxiety level (SMD: −0.42 [95% CI: −0.84; 0.003]; *p* = 0.052), though the pooled estimate was not statistically significant. The amount of heterogeneity was moderate (*I*^2^ = 70%) though statistically significant (*p* = 0.005).

To address potential heterogeneity sources, subgroup analysis, meta-regression, and leave-one-out analysis were performed.

**Figure 6:**
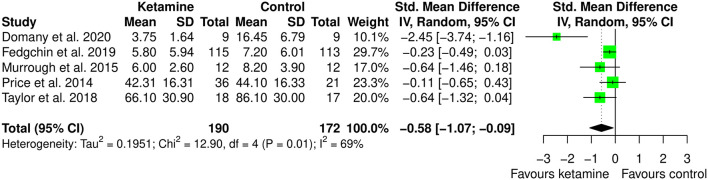
Subgroup meta-analysis of anxiety among ketamine/esketamine and control groups. SD, standard deviation; IV, inverse variance; 95% CI, 95% confidence interval. x axis labels have been edited according to the characteristics of the outcome: since lower scores indicate less anxiety the label “Favors ketamine” is on the left-hand side.

Subgroup meta-analysis was performed by removing the one study that used ketamine as adjuvant to ECT. This choice was made because ECT does not have any indication for anxiety, and it is an invasive treatment. We were interested in looking at potential different effects of ketamine when used as anesthetic within ECT session or as therapy. We believe that finding different effect for different administration type may be relevant since less invasive treatment may be better accepted by patients. Interestingly, the study that used ketamine within ECT session is the only one providing point estimate favoring controls, though with 95% CI crossing zero. Indeed, as displayed in Figure 6, in this meta-analysis ketamine (IV or intranasal) showed to be better than the comparators in the treatment of anxiety (SMD: −0.58 [95% CI: −1.07; −0.09]; *p* = 0.022). The heterogeneity estimate was still statistically significant (*I*^2^ = 69%; *p* = 0.012).

By inspecting Figure 5, showing the forest plot of the anxiolytic effect of ketamine, it is possible to observe that all but one studies have confidence intervals crossing zero. This is suggesting significant imprecision of the estimates across studies in the field, and potential bias in the pooled estimate due to outlier effects. Indeed, leave-one-out analysis, in which the meta-analysis of anxiety was serially repeated after the exclusion of each study, showed that by excluding the study from Domany et al. (2020) there is important decrease in heterogeneity (*I*^2^ = 23%), consistent with significant outlier effect, and a decrease of around 50% in the pooled estimate (SMD: −0.21 [95% CI: −0.46; 0.04]; *p* = 0.093). Irrelevant changes in the heterogeneity were obtained by excluding the other studies (leave-one-out data available in Supplementary Table 4).

Univariable and multivariable meta-regression analyses were performed on the following variables, potentially associated with heterogeneity: (1) depression effect size; (2) length of the follow-up (days); (3) age; (4) sex; (5) use (i.e., IV, intranasal spray, or adjuvant to ECT); (6) dose applied; (7) presence of treatment resistance. The depression effect size, the age of participants, and ketamine dose of 0.2 mg/kg resulted associated with the variance in anxiety at the univariable meta-regression model (unstandardized regression coefficient [B] = 2.59 [95% CI: 0.008; 5.17]; *p* = 0.049, *B* = 0.056 [95% CI: 0.009; 0.104]; *p* = 0.021, and *B* = −2.21 [95% CI: −3.71; −0.722]; *p* = 0.004, respectively). In the multivariable model age, esketamine dose of 56 mg, and ketamine dose of 0.2 mg/kg resulted as significant predictors of variance in anxiety above and beyond the effect on depression (age B = 0.035 [95% CI: 0.002; 0.068]; *p* = 0.039, esketamine 56 mg B = −1.85 [95% CI: −3.40; −0.294]; *p* = 0.020, ketamine 0.2 mg/kg B = −1.83 [95% CI: −3.20; −0.471]; *p* = 0.008; depression effect size B = −0.739 [95% CI: −3.84; 5.31]; *p* = 0.752, respectively). Multivariable model's *R*^2^ was 100%, meaning that 100% of the difference in true effect sizes can be explained by the set of predictors, which is quite substantial. Meta-regression results suggest that anxiety improves alongside depression during ketamine treatment, but this evidence is lost in the multivariable model where is evident that higher gain in anxiolytic effect is obtained at low ketamine dose (i.e., 0.2 mg/kg against 0.5 mg/kg) and in younger participants. However, given that the number of trials is <10, meta-regression results may be also biased from study with strong outlier effect (Hedges et al., 2011): this is the case of Domany et al. (2020), which was detected as significant outlier in leave-one-out analysis and is the only one that applied ketamine dose of 0.2 mg/kg. The results are displayed in Table 2.

**Table 2:** Meta-regression on anxiety ES.

**Variable(s)**	**B (95% CI)**	***p*-value**
Univariable analysis		
Depression ES	2.59 (0.008; 5.17)	0.049
Days of follow-up	0.026 (−0.011; 0.063)	0.165
Age	0.056 (0.009; 0.104)	0.021
% Females	−0.002 (−0.049; 0.045)	0.931
Administration type		
Adjuvant to ECT	0.162 (−1.22; 1.55)	0.819
IV treatment	−0.978 (−2.56; 0.604)	0.226
Spray treatment	−0.396 (-2.31; 1.52)	0.686
Dose applied		
Esketamine 56 mg	−0.234 (−0.798; 0.331)	0.417
Ketamine 0.2 mg/kg	−2.21 (−3.71; −0.722)	0.004
Ketamine 0.5 mg/kg	0.010 (−0.678; 0.698)	0.978
TRD	0.444 (−0.823; 1.71)	0.493
Multivariable analysis		
Age	0.035 (0.002; 0.068)	0.039
Depression ES	−0.739 (−3.84; 5.31)	0.752
Dose applied		
Esketamine 56 mg	−1.85 (-3.40; −0.294)	0.020
Ketamine 0.2 mg/kg	−1.83 (−3.20; −0.471)	0.008
Ketamine 0.5 mg/kg	0.074 (0.002; 0.068)	0.714

B, unstandardized linear regression coefficient; 95% CI, 95% confidence interval; ES, effect size; IV, intravenous; TRD, treatment resistant depression.

In addition, 2 more RCTs assessed the effect of ketamine on anxiety but did not provide quantitative data.

Zarate et al. (2012) randomized 15 subjects with bipolar (I or II) depression, maintained on therapeutic levels of lithium or valproate, to receive a single IV infusion of either ketamine (0.5 mg/kg) or placebo on 2 days 2 weeks apart. Subjects were repeatedly rated from 60 min before the infusion to 14 days post-infusion. The authors used the Hamilton Anxiety Rating Scale (HAM-A) and the Visual Analog Scale for Anxiety (VAS-A) to measure pre- and post-treatment anxiety levels, finding significant improvement of anxiety in patients who received ketamine from 40 min post-infusion, pointing out a rapid onset of action.

Zhong et al. (2016) enrolled and randomized 90 TRD patients to receive ketamine (0.8 mg/kg; *n* = 30), subanesthetic ketamine (0.5 mg/kg) plus propofol (0.5 mg/kg; *n* = 30) or propofol (0.8 mg/kg; *n* = 30) as adjuvant for ECT. Anxiety was measured with the Brief Psychiatric Rating Scale (BPRS-18) before the treatment and after 8 ECT sessions. The authors reported that patients in the ketamine group improved more than those in the ketamine plus propofol and the propofol only groups on the subscale of anxiety-depression.

### Effect of ketamine on quality of life and social functioning

Three studies (corresponding to 218 ketamine and 214 controls) provided outcome data on quality of life, measured with EuroQol visual analog scale (EQ-5D VAS), and 2 of these studies (corresponding to 187 ketamine and 178 controls) on social functioning, measured with Sheehan Disability Scale (SDS). The 2 trials which provided both data involved participants with MDD, used intranasal esketamine added on to current antidepressant treatment and placebo as comparator, the third trial analyzed for quality of life also involved participants with bipolar depression and compared ketamine and placebo as adjuvant to ECT.

**Figure 7:**
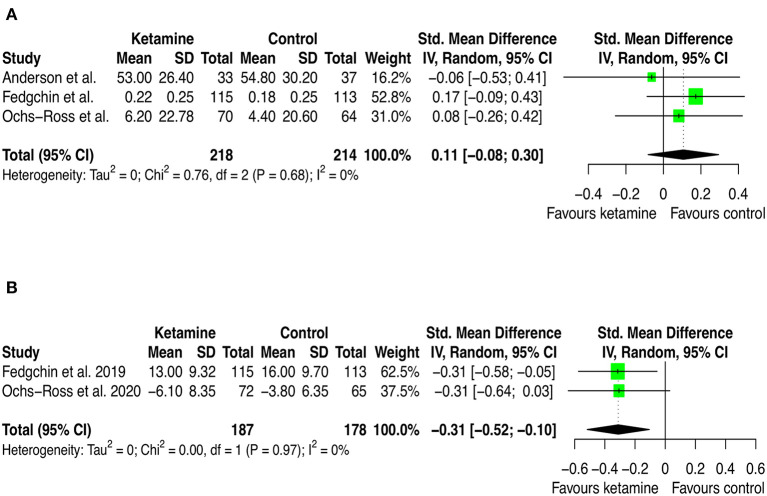
Forest plots of quality of life **(A)** and social functioning **(B)** among ketamine/esketamine and control groups. SD, standard deviation; IV, inverse variance; 95% CI, 95% confidence interval. x axis labels have been edited according to the characteristics of the outcome: since lower scores indicate better status the label “Favors ketamine” is on the left-hand side.

As can be seen in Figure 7 and Supplementary Table 3, ketamine was not different from the comparator in improving quality of life (SMD: 0.11 [95% CI: −0.08; 0.30]; *p* = 0.270), but intranasal esketamine was superior to placebo in improving social functioning (SMD: −0.31 [95%CI: −0.52; −0.10]; *p* = 0.003).

### Analysis of safety and tolerability

**Figure 8:**
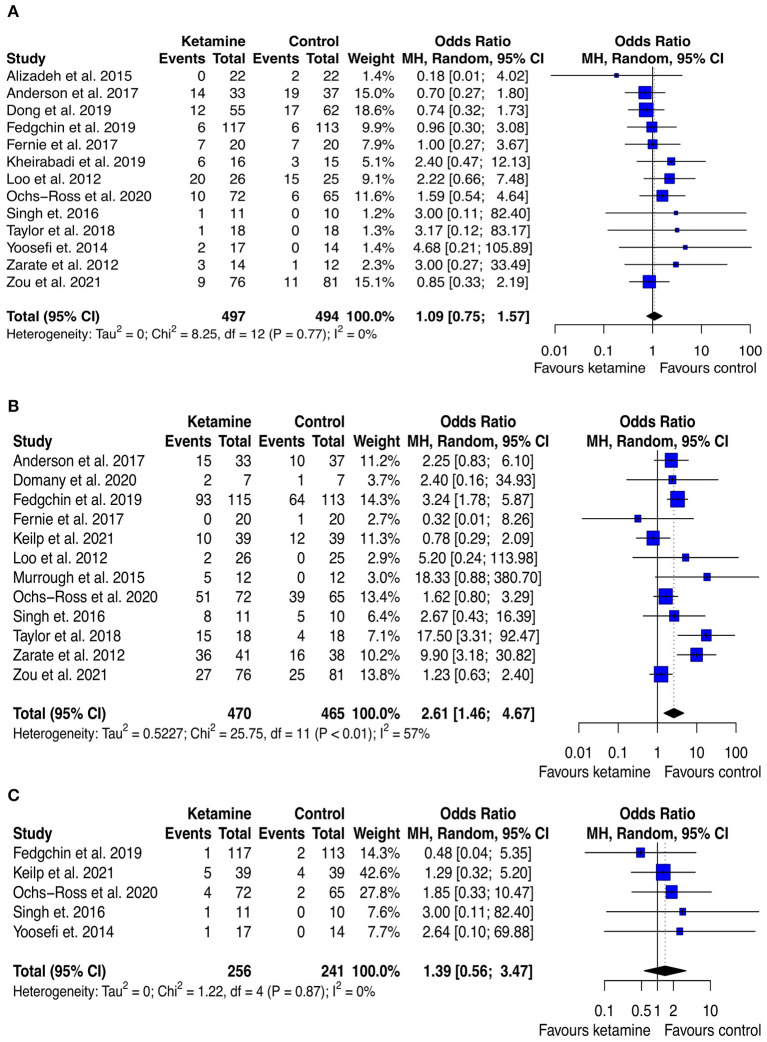
Forest plots of drop out due to any cause **(A)**, adverse effect **(B)**, and drop out due to serious adverse effect **(C)** among ketamine/esketamine and control groups. SD, standard deviation; MH, Mantel-Haenszel; 95% CI, 95% confidence interval. x axis labels have been edited according to the characteristics of the outcome: since lower rates indicate better safety/tolerability the label “Favors ketamine” is on the left-hand side.

The analysis of safety and tolerability of ketamine treatment was made by assessing the rates of adverse events, drop out due to any cause, and drop out due to serious adverse events across the experimental and control groups. The results are displayed in Figure 8 and Supplementary Table 5. The likelihood of adverse effects was higher among the ketamine treated group (pooled OR: 2.85 ([95% CI: 1.71; 4.76]; *p* < 0.001), however, the rates of drop out both due to any cause and to serious adverse effects were not significantly different across the study arms (pooled OR: 1.09 [95% CI: 0.75; 1.57]; *p* = 0.653, and pooled OR: 1.93 [95% CI: 0.86; −4.34]; *p* = 0.112, respectively). Notably, no death occurred both in the experimental and in the control groups of all the included trials.

### GRADE of the evidence

A detailed summary on the risk of bias in all 21 trials has been reported in the Appendix (see Supplementary Figures 2, 3), along with an assessment of the quality of the evidence (see Supplementary Table 6). In the GRADE system, the evidence from RCTs is initially set to high, there are then criteria that can be used either to downgrade or upgrade (see further information in the Appendix). The quality of the evidence is rated very low for most of the cognitive outcomes, low for anxiety, and moderate for quality of life and social functioning.

## Discussion

This systematic review and meta-analysis set out to investigate the effect of ketamine on trans-diagnostic psychopathology outcomes. In addition to depression, we were able to assess four different dimensions: cognition, anxiety, quality of life, and social functioning, alongside the assessment of the safety and tolerability of the interventions.

Our results suggest that ketamine treatment has an overall null effect on cognition, although the estimates are based on a small number of trials, and rather fragmented analyses due to the nature of the cognitive assessment that is made of many sub-dimensions. Nevertheless, this finding may still be relevant, considering that previous reports supported the negative effect of ketamine on cognition, especially in healthy volunteers and when used as drug of abuse (Krystal et al., 1994; Morgan et al., 2010). From the biochemical perspective, ketamine acts as a blocker of NMDA channels, thus leading to negative effects on cognition, at least theoretically. It is possible that the negative cognitive effects are mitigated by the improvement brought by the antidepressant effect of the drug, suggested by previous works (An et al., 2021; Bahji et al., 2021), and replicated in our analysis though with very low rating of the quality of the evidence. This hypothesis still requires confirmation, but it would suggest a possible pathway connecting depression and its cognitive symptoms (i.e., through NMDAR).

With respect to anxiety, this is the largest meta-analysis of ketamine intervention for anxiety symptoms. Our total sample size is larger than the one included in the previous meta-analysis of ketamine intervention in anxiety spectrum disorders (Whittaker et al., 2021) (6 vs. 2 RCTs, corresponding to 432 vs. 59 participants), and we confirmed the direction of the effect favoring ketamine. Differently from the work by Whittaker et al., we assessed anxiety symptoms trans-diagnostically, because anxiety is often comorbid with depression and other mental disorders. Although that led to an increased heterogeneity in the estimate, it allowed to include more studies and to support the hypothesis that ketamine may be effective in reducing anxiety regardless the categorical diagnosis. Indeed, when addressing the sources of heterogeneity through meta-regression techniques, we found that the improvement in the anxiety is mildly linked to the improvement in depression, supporting the hypothesis of the efficacy of ketamine on anxiety *per se*. Meta-regression also suggests that the anxiolytic effect of ketamine is more evident at low dose and among young people. The former evidence is contrary to available literature showing a dose-response profile for the anxiolytic effect of ketamine (Glue et al., 2017), and this inconsistency may be due to significant outlier effect in our analyses played by the study applying 0.2 mg/kg of ketamine. The stronger anxiolytic effect of ketamine among younger people instead, echoes previous evidence of better tolerability of ketamine (even at higher doses) and faster response to the treatment among young people (Di Vincenzo et al., 2021; Pennybaker et al., 2021). This different effect according to the age may ground on ketamine's pharmacological activity, which involves neuroplasticity pathways and long-term potentiation, that are attenuated with older age (Spriggs et al., 2017). Sub-group analysis revealed that the higher gain in the anxiolytic effect is obtained when ketamine is used as therapeutic agent rather than adjuvant to ECT. This is relevant and may warrant future RCTs to explore the use of ketamine in the treatment of anxiety. However, the moderate heterogeneity in the estimates and the marginally significant—statistically speaking—effect size, raise the question whether the effect is clinically relevant.

The pooled estimate on quality of life was not significant, whereas that on social functioning was, although based only on two studies. Arguably, the positive effect of ketamine treatment of social functioning reflects a change in the motivation, expressed as reduced anhedonia and disability linked to depression. If that will be confirmed by future, larger RCTs, it may be a very relevant effect of ketamine, considering the personal and social burden linked to depression.

Finally, in terms of safety and tolerability, ketamine treatment appears to be quite safe and accepted by the patients, as witnessed by the similar rates of drop out due to serious adverse effect or to any cause. Still, ketamine treatment is weighted by a higher rate of side effects, the most reported (i.e., with an incidence >5%) were not serious, with the notable exception of dissociation, and included vertigo, blurred vision, diarrhea, nausea, dizziness, and somnolence.

### Limitations

This review should be interpreted considering its limitations. First, cognition assessment has been performed rather differently across the included studies, reducing the comparability among them. That also translates in many meta-analyses (i.e., one for any cognitive domain), each including a small number of trials, with lack of statistical power in the analysis. Second, the dissociative effect of ketamine could have affected the blinding. For example, in one of the included trials, participants could identify when they were taking ketamine. This inadequate blinding has been quite typical in other saline-controlled (i.e., placebo) ketamine studies. For this reason, future trials should prefer use psychoactive comparators (e.g., midazolam). Third, the treatment with ketamine was used combined with antidepressants, and in many trials, it was not clear if simultaneous treatment with benzodiazepines was allowed. Concurrent use of benzodiazepines could be a relevant confounder both on cognitive and anxiety outcomes. Fourth, the domains of interest in our review were mostly collected as secondary outcomes in the trials, which could reduce the power and the robustness of the results (Jakobsen et al., 2019). Fifth, in the effort to be as comprehensive as possible and consistent with Cochrane guidelines (Chandler et al., 2008; Turner et al., 2008), we retrieved publicly available data outcomes from clinicaltrials.gov when these were not included in the final peer-review paper. The inclusion of these data is both a strength and a limitation of this review, as it allowed to mitigate possible publication bias, though the data collected may be of lower quality.

Sixth, the number of the included studies in each meta-analysis was <10, thus we could not inform about publication bias (Sterne et al., 2011).

Finally, we would like to remark that this is not a meta-analysis of ketamine for depression, which means that the estimate of depression effect-size is not based on a comprehensive list of all the reports on ketamine for depression. Despite this, we extracted depression outcome data from the included studies that reported it to assess with meta-regression if the change in depression levels may be important predictor of changes in the outcomes of interest for this review (i.e., cognition, anxiety, quality of life, and social functioning). The pooled effect-size of ketamine on depression favors ketamine, although the grading of this evidence was very low, mainly for detection of serious threats related to inconsistency. Indeed, 9 out of 17 (>50%) studies had SMD values crossing zero, which suggests a null effect within the frequentist framework.

### Implications for research and clinical practice

To the best of our knowledge, this is the first systematic review that assessed the effect of ketamine on cognition, quality of life, and social functioning. In addition, it is the largest meta-analysis of ketamine on anxiety. In summary, our findings appear to confirm the benefit of ketamine use in depression, also suggesting that ketamine may improve anxiety and social functioning, apparently without relevant negative effects on cognition.

The anxiolytic effect of ketamine seems to be quite independent from the antidepressant action, warranting future research investigating the effectiveness of the drug in the treatment of anxiety. These trials should ideally be designed to assess anxiety as the primary outcome, to have enough statistical power to detect relevant differences across the treatment and control groups. Alongside the replication of the anxiolytic effect, further investigation on how to combine ketamine treatment with psychosocial interventions may have relevant clinical implications. Adaptation of psychological treatments which showed to have impacts on anxiety, for example cognitive-behavioral therapy (CBT) approaches (Carpenter et al., 2018), with ketamine, could provide a basis for the development of new treatment for anxiety.

According to Cohen's suggestion (Cohen, 1988), the effect size for the anxiolytic effect of ketamine ranged from moderate to small. Following Furukawa's method for the calculation of NNT from Cohen's d (Furukawa, 1999; Furukawa and Leucht, 2011), our results mean that around 5 patients should be treated with ketamine to have one who improves ≥50% in anxiety level. Concerning any adverse effects, the NNH of ketamine treatment was 6. It should be noted that ketamine treatment is now delivered to the most severe patients, such as treatment-resistant or suicidal, making the estimate of the effect size rather impressive for such a complex population. In addition, these patients usually suffer also from worst physical health (Chan et al., 2022; Marchi et al., 2022). Future trials should try to assess if ketamine treatment may be better tolerated by people without such complexity and high levels of physical frailty, also implementing lower doses arms to assess if similar outcomes may be obtained also at lower doses and with lower side effects. So far, in the absence of such data, when starting treatment with ketamine evaluation of the possible benefits/harms should be taken carefully.

Finally, our grading of the evidence ranged from moderate to very low, with serious threats detected by the risk of bias assessment. We highlight the need for a more precise design of the studies and stricter adherence to guidelines on the reporting of trial results.

## Conclusions

Overall, our findings suggest that ketamine treatment may improve anxiety and social functioning of adults with affective disorders. The anxiolytic effect appears to be at least partly independent of the antidepressant action, warranting future research on the use of ketamine in the treatment of anxiety. Given the concurrent high rate of adverse events during the ketamine treatment, future trials should also focus on better understanding how to balance pros and cons of the treatment.

## Data availability statement

The codes for reproducing the datasets and the analyses can be accessed here: https://github.com/MattiaMarchi/Meta-Analysis---Ketamine-multidimensional-psychopathology. Further inquiries can be directed to the corresponding author.

## Author contributions

Conceptualization and planning: MM, GG, FMM, AT, DU, ED, and FM. Acquisition: MM, GG, FMM, AT, DU, ED, and FM. Analysis of the data: MM. Interpretation of the results: MM, GG, FMM, AT, DU, ED, FM, and GMG. Drafting: MM, GG, FMM, AT, DU, ED, and FM. Critical revision of the manuscript: SF, LP, and GMG. All authors approved the final submitted version of the manuscript.

## Conflict of interest

The authors declare that the research was conducted in the absence of any commercial or financial relationships that could be construed as a potential conflict of interest.

## Publisher's note

All claims expressed in this article are solely those of the authors and do not necessarily represent those of their affiliated organizations, or those of the publisher, the editors and the reviewers. Any product that may be evaluated in this article, or claim that may be made by its manufacturer, is not guaranteed or endorsed by the publisher.
