# Supplementary material for: The effect of ketamine on cognition, anxiety, and social functioning in adults with psychiatric disorders: A systematic review and meta-analysis
Source: Front Neurosci. 2022 Nov 24;16:1011103. doi: 10.3389/fnins.2022.1011103 (PMC9730521; doi:10.3389/fnins.2022.1011103)
Supplement: Supplementary file 1 [file Data_Sheet_1.docx]

**Appendix - Supplementary Materials**

Supplementary Table 1: Search strategy and number of hits per database (current to 30^th^ April 2022).

| **Database** | **String** | **Number of records** |
| --- | --- | --- |
| PubMed (Medline) | ((("Ketamine"[Mesh]) OR ("Esketamine" [Supplementary Concept])) AND ("Mental Disorders"[Mesh])) AND ("Randomized Controlled Trial" [Publication Type]) | 309 |
| Scopus | ( ( TITLE-ABS-KEY ( ketamine ) OR TITLE-ABS-KEY ( esketamine ) ) ) AND ( TITLE-ABS-KEY ( mental AND disorders ) ) AND ( TITLE-ABS-KEY ( randomized AND controlled AND trials ) ) | 187 |
| EMBASE | ('mental disease'/exp OR 'abnormal mental state' OR 'disease, mental' OR 'diseased mental state' OR 'disorder, mental' OR 'disordered mental state' OR 'disturbed mental state' OR 'illness, mental' OR 'insanity' OR 'mental abnormality' OR 'mental change' OR 'mental confusion' OR 'mental defect' OR 'mental disease' OR 'mental disorder' OR 'mental disorders' OR 'mental disorders diagnosed in childhood' OR 'mental disturbance' OR 'mental illness' OR 'mental insufficiency' OR 'mental symptom' OR 'mentally ill' OR 'neurodevelopmental disorder' OR 'neurodevelopmental disorders' OR 'neuropsychiatric disease' OR 'neuropsychiatric diseases' OR 'neuropsychiatric disorder' OR 'neuropsychiatric disorders' OR 'psychiatric disease' OR 'psychiatric disorder' OR 'psychiatric illness' OR 'psychiatric symptom' OR 'psychic disease' OR 'psychic disorder' OR 'psychic disturbance' OR 'psychologic disorder' OR 'psychologic disturbance' OR 'psychological disorder' OR 'psychological disturbance' OR 'psychopathology') AND ('ketamine'/exp OR '2 (2 chlorophenyl) 2 (methylamino) cyclohexanone' OR '2 (2 chlorophenyl) 2 (methylamino) cyclohexanone hydrochloride' OR '2 (2 chlorophenyl) 2 methylaminocyclohexanone' OR '2 (methylamino) 2 (2 chlorophenyl) cyclohexanone' OR '2 (ortho chlorophenyl) 2 (methylamino) cyclohexanone' OR '2 (ortho chlorophenyl) 2 methylaminocyclohexanone' OR '2 (ortho chlorophenyl) 2 methylaminocyclohexanone hydrochloride' OR '2 methylamino 2 (2 chlorophenyl) cyclohexanone' OR '2 ortho chlorophenyl 2 methylaminocyclohexanone' OR 'anesject' OR 'calipsol' OR 'calypsol' OR 'ci 581' OR 'ci581' OR 'cl 369' OR 'cl369' OR 'cn 52, 372 2' OR 'cn 52372 2' OR 'cn 523722' OR 'cn52, 372 2' OR 'cn52372 2' OR 'cn523722' OR 'ereska' OR 'imalgene' OR 'kalipsol' OR 'katamine' OR 'keta-hameln' OR 'ketaject' OR 'ketalar' OR 'ketalin' OR 'ketamax' OR 'ketamine' OR 'ketamine hcl' OR 'ketamine hydrochloride' OR 'ketaminol vet' OR 'ketanest' OR 'ketased' OR 'ketaset' OR 'ketaved' OR 'ketavet' OR 'ketmin' OR 'ketoject' OR 'ketolar' OR 'narkamon' OR 'narketan' OR 'pmi 100' OR 'pmi 150' OR 'pmi100' OR 'pmi150' OR 'sls 002' OR 'sls002' OR 'soon-soon' OR 'tekam' OR 'tur 002' OR 'tur002' OR 'velonarcon' OR 'vetalar' OR 'esketamine'/exp OR 'am 101' OR 'am101' OR 'esketamine' OR 'esketamine hydrochloride' OR 'falkieri' OR 'jnj 54135419' OR 'jnj 5419' OR 'jnj54135419' OR 'jnj5419' OR 'ketanest s' OR 'keyzilen' OR 'pg 061' OR 'pg061' OR 's ketamin' OR 's ketamine' OR 's-ketamin' OR 'spravato' OR 'vesierra') AND ('cognition'/exp OR 'cognition' OR 'cognitive accessibility' OR 'cognitive balance' OR 'cognitive dissonance' OR 'cognitive function' OR 'cognitive structure' OR 'cognitive symptoms' OR 'cognitive task' OR 'cognitive thinking' OR 'neurobehavioural manifestations' OR 'volition' OR 'social psychology'/exp OR 'psychology, social' OR 'psychosocial adjustment' OR 'psychosocial aspect' OR 'psychosocial factor' OR 'psychosocial functioning' OR 'psychosocial stability' OR 'psychosocial study' OR 'psychosociality' OR 'psychosociology' OR 'social conformity' OR 'social psychology' OR 'sociopsychology' OR 'quality of life'/exp OR 'hrql' OR 'health related quality of life' OR 'life quality' OR 'quality of life' OR 'anxiety'/exp OR 'anxiety' OR 'social phobia'/exp OR 'phobia, social' OR 'social anxiety disorder' OR 'social phobia' OR 'sociophobia') AND ('randomized controlled trial'/exp OR 'controlled trial, randomized' OR 'randomised controlled study' OR 'randomised controlled trial' OR 'randomized controlled study' OR 'randomized controlled trial' OR 'trial, randomized controlled') | 565 |
| PsycINFO | **(**mental health or mental illness or mental disorder or psychiatric illness) AND (MA esketamine OR MA ketamine) AND (TX randomised controlled trial or randomized controlled trial or rct) | 14 |

Supplementary Figure 1: Forest plots of depression among ketamine/esketamine and control groups.

Abbreviations: SD: standard deviation; IV: inverse variance; 95% CI: 95% confidence interval.

Legend: x axis labels have been edited according to the characteristics of the outcome: since lower scores indicate less anxiety the label “Favours ketamine” is on the left-hand side.

Supplementary Table 2: Meta analysis of the cognitive outcomes

| **Outcome** | **N studies** | **N Ketamine/ Controls** | **SMD (95% CI)** | **p-value** | **I^2^ (p-value)** |
| --- | --- | --- | --- | --- | --- |
| AMI-SF | 2 | 55/61 | -0.07 (-0.44; 0.29) | 0.701 | 0% (0.778) |
| Attention | 2 | 115/99 | 0.65 (-0.83; 2.12) | 0.390 | 96% (<0.001) |
| Category Fluency | 3 | 94/100 | -0.51 (-1.31; 0.29) | 0.215 | 86% (0.001) |
| Copy | 2 | 55/61 | 0.02 (-0.74; 0.77) | 0.967 | 75% (0.045) |
| Delayed Recall | 4 | 166/165 | -0.40 (-1.07; 0.28) | 0.249 | 88% (<0.001) |
| Immediate Recall | 3 | 94/100 | -0.26 (-0.85; 0.32) | 0.382 | 75% (0.018) |
| Letter Fluency | 3 | 94/100 | -0.79 (-1.79; 0.21) | 0.123 | 91% (<0.001) |
| MMSE | 2 | 84/89 | 0.08 (-0.22; 0.38) | 0.605 | 0% (0.596) |
| Recognition Discrimination | 3 | 94/100 | -0.30 (-0.72; 0.13) | 0.174 | 54% (0.112) |
| Retention | 2 | 55/61 | -0.12 (-0.48; 0.25) | 0.527 | 0% (0.653) |
| Total Learning | 3 | 127/126 | -0.40 (-0.65; -0.15) | 0.002 | 0% (0.609) |
| Visual Learning | 2 | 115/99 | -0.04 (-0.56; 0.47) | 0.875 | 70% (0.066) |
| Working Memory | 2 | 115/99 | -0.02 (-0.29; 0.24) | 0.862 | 0% (0.815) |
| CANTAB-SRM | 1 | 20/20 | 0.43 (-0.20; 1.05) | 0.184 | NA |
| Executive Functions | 1 | 72/65 | 0.25 (-0.09; 0.58) | 0.149 | NA |
| Post-ECT Cognitive Recovery Time | 1 | 22/20 | -0.53 (-1.14; 0.09) | 0.094 | NA |
| MoCA | 1 | 43/45 | 0.18 (-0.24; 0.60) | 0.401 | NA |
| Social Cognition | 1 | 43/34 | 0.39 (-0.07; 0.84) | 0.094 | NA |
| Verbal Learning | 1 | 43/34 | -0.18 (-0.63; 0.27) | 0.429 | NA |
| WMS | 1 | 16/15 | 0.20 (-0.51; 0.91) | 0.578 | NA |

Abbreviations: SMD: standardized mean difference; 95% CI: 95% confidence interval; AMI-SF: Autobiographical memory interview short form; MMSE: mini-mental state examination; CANTAB-SRM: Cambridge Automated Neuropsychological Test Battery Spatial Recognition Memory task; MoCA: Montreal cognitive assessment; WMS: Wechsler Memory Scale; NA: where the estimate is based on the results of 1 trial, the heterogeneity test is not meaningful.

Legend: except for post-ECT cognitive recovery time where lower scores indicate better performance, for all the other instruments higher scores indicate better performance.

Supplementary Table 3: Meta analysis of the secondary outcomes

| **Outcome** | **N studies** | **N Ketamine/ Controls** | **SMD (95% CI)** | **p-value** | **I^2^ (p-value)** |
| --- | --- | --- | --- | --- | --- |
| Anxiety | 6 | 223/209 | -0.42 (-0.84; 0.003) | 0.052 | 70% (0.005) |
| QoL | 3 | 218/214 | 0.11 (-0.08; 0.30) | 0.270 | 0% (0.683) |
| Social Functioning | 2 | 187/178 | -0.31 (-0.52; -0.10) | 0.003 | 0% (0.970) |
| Depression | 16 | 595/583 | -0.78 (-1.20; -0.35) | <0.001 | 91% (<0.001) |

Abbreviations: SMD: standardized mean difference; 95% CI: 95% confidence interval; QoL: quality of life.

Supplementary Table 4: Leave-one-out analysis of anxiety

| **Study ID** | **SMD (95% CI)** | **p-value** | **I^2^** |
| --- | --- | --- | --- |
| Anderson et al. 2017 | -0.603 (-1.11; -0.093) | 0.024 | 72% |
| Domany et al. 2020 | -0.210 (-0.455; 0.035) | 0.093 | 23% |
| Fedgchin et al. 2019 | -0.576 (-1.22; 0.066) | 0.078 | 77% |
| Murrough et al. 2015 | -0.421 (-0.911; 0.069) | 0.092 | 76% |
| Price et al. 2014 | -0.552 (-1.09; -0.009) | 0.046 | 77% |
| Taylor et al. 2018 | -0.417 (-0.918; 0.083) | 0.102 | 76% |

Abbreviations: SMD: standardized mean difference; 95% CI: 95% confidence interval.

Supplementary Table 5: Meta analysis of safety/tolerability outcomes

| **Outcome** | **N studies** | **N Ketamine/ Controls** | **OR (95% CI)** | **p-value** | **I^2^ (p-value)** |
| --- | --- | --- | --- | --- | --- |
| Drop out due to any cause | 12 | 497/494 | 1.09 (0.75; 1.57) | 0.653 | 0% (0.776) |
| Adverse events | 12 | 470/465 | 2.61 (1.46; 4.67) | 0.001 | 57% (0.007) |
| Drop out due to adverse event | 5 | 256/241 | 1.39 (0.56; 3.47) | 0.476 | 0% (0.875) |

Abbreviations: OR: odds ratio; 95% CI: 95% confidence interval

Supplementary Figure 2: Risk of bias graph showing review authors' judgements about each risk of bias item presented as percentages across all included studies

Supplementary Figure 3: Risk of bias summary showing review authors' judgements about each risk of bias item for each included study.

Supplementary Table 6: GRADE evidence summary for each outcome

| **Certainty assessment** | | | | | | | **N° of patients** | | **Effect** | **Certainty** | **Importance** |
| --- | --- | --- | --- | --- | --- | --- | --- | --- | --- | --- | --- |
| **N° of studies** | **Study design** | **Risk of bias** | **Inconsistency** | **Indirectness** | **Imprecision** | **Other considerations** | **Ketamine** | **Controls** | **SMD**  **(95% CI)** |  |  |
| *AMI-SF* | | | | | | | | | | | |
| 2 | RCTs | Serious | Not Serious | Not serious | Serious | Serious | 55 | 61 | -0.07  (-0.44; 0.29) | +???  VERY LOW | Not important |
| *Attention* | | | | | | | | | | | |
| 2 | RCTs | Serious | Serious | Not serious | Serious | Serious | 115 | 99 | 0.65  (-0.83; 2.12) | ????  VERY LOW | Not important |
| *Category Fluency* | | | | | | | | | | | |
| 3 | RCTs | Serious | Serious | Not serious | Serious | Not serious | 94 | 100 | -0.51  (-1.31; 0.29) | +???  VERY LOW | Not important |
| *Copy* | | | | | | | | | | | |
| 2 | RCTs | Serious | Serious | Not serious | Serious | Serious | 55 | 61 | 0.02  (-0.74; 0.77) | +???  VERY LOW | Not important |
| *Delayed Recall* | | | | | | | | | | | |
| 4 | RCTs | Serious | Serious | Not serious | Serious | Not serious | 166 | 165 | -0.40  (-1.07; 0.28) | +???  VERY LOW | Not important |
| *Immediate Recall* | | | | | | | | | | | |
| 3 | RCTs | Serious | Serious | Not serious | Serious | Not serious | 94 | 100 | -0.26  (-0.85; 0.32) | +???  VERY LOW | Not important |
| *Letter Fluency* | | | | | | | | | | | |
| 3 | RCT | Serious | Serious | Not serious | Serious | Not serious | 94 | 100 | -0.79  (-1.79; 0.21) | +???  VERY LOW | Not important |
| *MMSE* | | | | | | | | | | | |
| 2 | RCTs | Serious | Not serious | Not serious | Serious | Serious | 84 | 89 | 0.08  (-0.22; 0.38) | +???  VERY LOW | Not important |
| *Recognition Discrimination* | | | | | | | | | | | |
| 3 | RCTs | Serious | Not serious | Not serious | Serious | Not serious | 94 | 100 | -0.30  (-0.72; 0.13) | ++??  LOW | Not important |
| *Retention* | | | | | | | | | | | |
| 2 | RCTs | Serious | Not serious | Not serious | Serious | Serious | 55 | 61 | -0.12  (-0.48; 0.25) | +???  VERY LOW | Not important |
| *Total Learning* | | | | | | | | | | | |
| 3 | RCTs | Serious | Not serious | Not serious | Not serious | Not serious | 127 | 126 | -0.40  (-0.65; -0.15) | +++?  MODERATE | Important |
| *Visual Learning* | | | | | | | | | | | |
| 2 | RCTs | Serious | Not serious | Not serious | Serious | Serious | 115 | 99 | -0.04  (-0.56; 0.47) | +???  VERY LOW | Not important |
| *Working Memory* | | | | | | | | | | | |
| 2 | RCTs | Serious | Not serious | Not serious | Serious | Serious | 115 | 99 | -0.02  (-0.29; 0.24) | +???  VERY LOW | Not important |
| *Anxiety* | | | | | | | | | | | |
| 6 | 5 RCTs/1 crossover RCT | Serious | Not serious | Not serious | Serious | Not serious | 223 | 209 | -0.42  (-0.84; 0.001) | ++??  LOW | Important |
| *Quality of Life* | | | | | | | | | | | |
| 3 | RCTS | Not serious | Not serious | Not serious | Serious | Not serious | 218 | 214 | 0.11  (-0.08; 0.30) | +++?  MODERATE | Important |
| *Social Functioning* | | | | | | | | | | | |
| 2 | RCTs | Not serious | Not serious | Not serious | Not serious | Serious | 187 | 178 | -0.31  (-0.52; -0.10) | +++?  MODERATE | Important |
| *Depression* | | | | | | | | | | | |
| 16 | 15 RCTs/1 crossover RCT | Serious | Serious | Not serious | Not serious | Not serious | 595 | 583 | -0.78  (-1.20; -0.35) | +???  VERY LOW | Important |

Abbreviations: SMD: standardized mean difference; 95% CI: 95% confidence interval; AMI-SF: Autobiographical memory interview short form; RCT: randomized controlled clinical trial; MMSE: mini-mental state examination.

*Explanation of reasons for downgrading/upgrading:*

We GRADEd each pooled estimate for each relevant outcome according to the following criteria:

a. ***Risk of Bias:*** *We downgraded this domain by one level when any of the sources of Risk of Bias (as described above) were rated as “high”, or by half level when rated as “unclear”, for any of the studies included in the pooled estimate.*

b. ***Inconsistency:*** *We downgraded this domain by one level where the I^2^ value indicated substantial levels of heterogeneity (i.e., I^2^≥75%).*

c. ***Indirectness:*** *We protected against indirectness in this review by ensuring all studies included in any meta-analysis reported data from a validated psychometric tool. We therefore did not downgrade this domain for any assessment point.*

d. ***Imprecision:*** *We downgraded this domain by one level where the 95% confidence interval included the null value.*

e. ***Other Considerations:*** *We downgraded by one level when the number of studies contributing to the pooled estimate was ≤2.*
